# Prevalence and correlates of anxiety and depression in frontline healthcare workers treating people with COVID-19 in Bangladesh

**DOI:** 10.1186/s12888-021-03243-w

**Published:** 2021-05-25

**Authors:** Rafia Tasnim, Md. Safaet Hossain Sujan, Md. Saiful Islam, Asmaul Husna Ritu, Md. Abid Bin Siddique, Tanziha Yeasmin Toma, Rifat Nowshin, Abid Hasan, Sahadat Hossain, Shamsun Nahar, Salequl Islam, Muhammad Sougatul Islam, Marc N. Potenza, Jim van Os

**Affiliations:** 1 Department of Public Health and Informatics, Jahangirnagar University, Savar, Dhaka, 1342 Bangladesh; 2 Centre for Advanced Research Excellence in Public Health, Savar, Dhaka, 1342 Bangladesh; 3 Department of Population Science and Human Resource Development, Rajshahi University, Rajshahi Sadar, Rajshahi, 6205 Bangladesh; 4 Department of Microbiology, Jahangirnagar University, Savar, Dhaka, 1342 Bangladesh; 5 BioTED, Mirpur, Dhaka, 1216 Bangladesh; 6 Department of Psychiatry and Child Study Center, Yale School of Medicine, New Haven, Connecticut USA; 7 Connecticut Mental Health Center, New Haven, Connecticut USA; 8 Connecticut Council on Problem Gambling, Wethersfield, Connecticut USA; 9 Department of Neuroscience, Yale University, New Haven, Connecticut USA; 10 Department of Psychiatry, UMC Utrecht Brain Center, University Medical Center Utrecht, Utrecht University, Utrecht, The Netherlands; 11 Department of Psychosis Studies, Institute of Psychiatry, Psychology & Neuroscience, King’s College London, London, United Kingdom

**Keywords:** Anxiety, Depression, Healthcare workers, COVID-19, Bangladesh

## Abstract

**Background:**

Healthcare workers (HCWs) who are in the frontline during the COVID-19 pandemic are often under significant pressures that may predispose them to symptoms of poor mental health. This study aimed to investigate the prevalence of anxiety and depression among HCWs and factors correlated with mental health concerns during the COVID-19 pandemic in Bangladesh. And, it also aimed to evaluate the psychometric properties of the Bangla version of the Hospital Anxiety and Depression Scale (HADS).

**Methods:**

A cross-sectional survey was conducted between July and August, 2020. A self-reported online questionnaire was utilized to collect data. The survey included questions concerning socio-demographic, lifestyle, and work setting, as well as the HADS. A confirmatory factor analysis (CFA) and multiple linear regression analysis were performed.

**Results:**

Data from 803 HCWs (50.7% male; mean age: 27.3 [SD = 6.9]; age range: 18-58 years) were included in the final analysis. The Bangla HADS was psychometrically sound, and demonstrated good internal consistency and reliability (α = 0.83), and excellent construct validity. Prevalence estimates of anxiety and depression were 69.5%, and 39.5%, respectively, for less severe symptomology (at least borderline abnormal), and 41.2% and 15.7% for more severe (at least abnormal) symptomology. Regression analyses with the total HADS score as a dependent variable revealed significant (*p *< 0.05) associations with female gender, moderate and poor health status, infrequent physical exercising, smoking, having had regrets about one’s profession because of the pandemic and associated experiences, not updating on the latest COVID-19-related research, experiencing discrimination in the workplace, and facing social problems due to working in a lab or hospital during the COVID-19 pandemic.

**Conclusions:**

Symptoms of anxiety and depression are prevalent among HCWs during the COVID-19 pandemic in Bangladesh. The findings suggest a need for screening for mental health concerns, and employing early intervention to help these individuals.

## Background

The emergence of a novel coronavirus, severe acute respiratory syndrome coronavirus-2 (SARS CoV-2), which causes coronavirus disease 2019 (COVID-19), has substantially impacted healthcare systems globally. The COVID-19 pandemic has generated a public health crisis worldwide [1]. The World Health Organization (WHO) declared it an international public health emergency [2]. According to the WHO, there have been over 31.1 million confirmed cases of COVID-19, including 962,008 deaths as of September 22, 2020 [3]. Globally, there have been more deaths from COVID-19 than from severe acute respiratory syndrome (SARS) and Middle East respiratory syndrome (MERS) combined, although there has been a relatively lower death rate for COVID-19 among people infected with SARS CoV-2 [4]. Currently, no fully effective treatment has been developed for treating patients with COVID-19 [5]. This global pandemic poses a huge challenge for local healthcare services. As the number of patients with COVID-19 increases, health resources, including inpatient or intensive care beds, ventilators, medications, and personal protective equipment (PPE), have at times been limited. With fewer resources, health care workers (HCWs) may experience considerable pressure and anxiety [1, 6].

Frontline HCWs during the COVID-19 pandemic may be at considerable risk of developing mental problems [7, 8], as HCWs were during SARS or Ebola outbreaks [9]. Frontline HCWs directly involved in diagnosing and treating COVID-19 patients may experience mental distress and other mental health concerns [7, 10]. The large number of confirmed and suspected cases, disproportionate workloads, shortages of PPE and medications, extensive media attention, and feelings of insufficient assistance may predispose to emotional strain in HCWs [10–12]. Prior studies have reported that HCWs feared for contagion and contamination of their family, friends, and colleagues [13], felt insecurity and shame, reported unwillingness to work or contemplate resignation [14], and frequently reported stress, anxiety, and depression [15, 16]. Thus, understanding shorter- and longer-term mental impacts on HCWs is important.

During the SARS epidemic, 29%-35% of HCWs experienced considerable distress [17]. Several years after the outbreak, 10% of healthcare staff reported symptoms of post-traumatic stress disorder (PTSD) [18]. Individuals who had experienced quarantines or served in wards treating infected patients were two- or three-fold more likely to experience PTSD [18].

Timely interventions are important for improving the mental resilience of HCWs and optimizing function of healthcare systems [19]. Effective communication, restriction of shift hours, provision of rest areas and broad access to them, availability and appropriate management of PPE, and advanced training on COVID-19 may minimize HCWs’ anxiety [20].

Bangladesh, where the present study was conducted, is a South Asian country whose healthcare system has been considerably impacted by COVID-19. Bangladesh has experienced numerous COVID-19 patients, lack of preparedness, and shortages of hospital beds and other items relevant to COVID-19 patient care. In Bangladesh, the virus was first reported on March 8, 2020 [21–23]. Since then, the virus quickly spread and there have now been more than 352,178 cases and 5,007 deaths at the time of writing (September 22, 2020) [24]. According to recent reports of the Bangladesh Medical Association, there were 8,125 infected cases of HCWs including doctors, nurses, and other staff with 117 fatalities (115 physicians and 2 dental surgeons) [25, 26]. Moreover, there is also an acute shortage of medical practitioners, including physicians, nurses and medical personnel in this country. Bangladesh has 0.79 hospital bed per 10,000 population, while China has 4.31, and the USA has 2.87 beds per 1,000 people [27]. Moreover, the government health department of Bangladesh has only 432 ICU beds in total (total population 170 million), only 110 of which are placed outside the capital city, Dhaka. The private healthcare sector has an additional 737 ICU beds [28, 29]. The Bangladesh government has published an act describing the rules of infectious disease management and the allocation of staff; it declared an emergency response committee to monitor the spread of the infection [30]. The number of HCWs is insufficient as the number of positive patients has been rising rapidly, overwhelming HCWs who are in contact with these positive patients. To reduce the transmission, the government published a plan, identifying six basic country-wide response measures. The government also implemented a plan to form a 500-person local response committee covering the country along with the national Rapid Response Committee (RRC) and the Rapid Response Team (RRT). Additionally, 500 hospital beds were prepared for the treatment of COVID patients [31]. Hence, the government of Bangladesh took important steps, and applied these at a national level for the prevention of transmission of the virus [32], although there exist various macro or microsystemic reasons for why the WHO recommendations sometimes cannot be fully followed [33].

During the pandemic, HCWs have been experiencing heavy workloads and shortages of PPE, and these and other factors may increase anxiety [6, 34]. Shortcomings in COVID-19 testing facilities may worsen the situation [35]. There is no specific working schedule for HCWs and sometimes they are required to work up to 17 hours per day [36]. In this study, respondents (HCWs) completed the questionnaire after at least several months of frontline exposure during the pandemic.

The impact of COVID-19 may be influenced significantly by psychosocial and cultural contexts. While differential impact of COVID in higher-income and lower-income countries have been hypothesized, few studies have investigated COVID-19-related phenomena in lower-income countries. It is therefore important to gather COVID-19-related data in countries with limited resources like Bangladesh in order to understand mental health concerns and related factors. It is particularly important to understand these concerns in HCWs in under-resourced countries as changes in mental health often have long-term consequences, potentially furthering the impact of COVID-19 on HCWs after the pandemic subsides. In addition, mental health impacts on HCWs due to the COVID-19 have been poorly studied in Asian jurisdictions and lower-income countries like Bangladesh. We hypothesized that many HCWs would report anxiety and depression and, as in prior outbreaks, anxiety and depression would be associated with virus-related experiences and exposures.

### Study objectives

The study objectives were to investigate mental health among Bangladeshi HCWs treating COVID-19 patients by assessing severity of depression and anxiety; to assess relationships between depression and anxiety and care-related measures; and, to evaluate the psychometric properties of the Bangla version of the Hospital Anxiety and Depression Scale (HADS).

## Methods

### Participants and procedure

A cross-sectional survey was conducted among HCWs (e.g., doctors, nurses, public health professionals, lab workers, and other caregivers) during the COVID-19 pandemic in Bangladesh. Here, public health professionals (e.g., epidemiologists, biostatisticians, health educators) are those who had direct interaction with COVID-19 patients, and whose role formed part of the national plan to alleviate the spread of COVID-19. Lab workers were those people who are working in the lab to identify individuals' COVID-19 status (positive/negative). Other caregivers included pharmacologists, microbiologists, virologists, and biotechnologists. All participants (HCWs) had full to limited, but at least some, direct interaction with COVID-19 patients in their workplace. Data were collected between July and August, 2020, using a self-reported online survey. After administering all survey questions in Google Forms, a shareable link was generated. This link was then shared in different online platforms involving HCWs to collect data efficiently in the setting of lockdown and spatial distancing measures. Individuals provided informed consent prior to participating in the study. Approximately 8-10 minutes were required to complete the entire questionnaire. The inclusion criteria included (i) 18 years or older; and (ii) completing the entire questionnaire. Initially, 825 respondents participated. Ultimately, 803 surveys (50.7% males; mean age: 27.3 years [SD = 6.9]; age range: 18-58 years) were included in final analysis after removing incomplete surveys.

### Measures

A self-reported survey questionnaire assessed variables related to socio-demographics, lifestyle, work environment, anxiety, and depression. Variables selection was guided by previous epidemiological literature on anxiety and depression in Bangladesh [37–40].

#### Socio-demographic and lifestyle measures

Socio-demographic information included gender, age, marital status, occupation, and residence location (urban/rural). Self-reported health status (good, moderate, and poor) and chronic diseases (e.g., high blood pressure, heart problems, diabetes, respiratory problems; yes/no) were also assessed. Numbers of average sleep hours, physical exercising (yes/no), and current smoking (yes/no) were assessed. Sleeping status, conforming to methodology in previous works in the Bangladeshi population [38, 39, 41, 42], was classified into three categories as previously: normal [7-9 hours], less than normal [< 7h], and more than normal [> 9h].

#### Work-setting-related questions

The following ‘yes/no’ questions concerning the working environment were asked during the survey: *Have you been infected with COVID-19 while at work?*, *Have any of your colleagues been infected with COVID-19?*, *Do you feel adequately trained to conduct COVID-19-related treatment/PCR tests/provision of patient care?*, *Have you experienced shortages of PPE/hand sanitizer/masks during the pandemic?*, *Have you had regrets about your choice of profession because of the pandemic and related unexpected experiences?*, *Are you frequently updating yourself with the latest research papers on COVID-19?*, *Do you currently have to work overtime?, Have you ever been discriminated against in your workplace (lab or hospital)?*, *Have you been counseled regarding how to maintain your mental health in the current situation?*, and *Have you encountered social problems (e.g., neighbor neglect) due to working in a lab or hospital?*

#### Hospital Anxiety and Depression Scale (HADS)

The HADS is a self-reported instrument for assessing anxiety and depression symptomatology. The HADS was developed by Zigmond & Snaith (1983) which is widely used globally. This scale consists of 14 questions concerning problems over the past week (e.g., “*I still enjoy the things I used to enjoy*”) including 2 subscales (i.e., 7-item anxiety and 7-item depression) with a four-point Likert scale ranging from 0 to 3 [43]. The raw scores for each subscale are summed as total scores ranging from 0 to 21 for each subscale. The present study employed the Bangla HADS described recently [44]. The most widely used standardized translation procedure, proposed by Beaton et al. (2000), was used to perform the back translation for this scale [45]. In the present study, predefined thresholds for normal (0–7), borderline abnormal (8-10), and abnormal (11-21) levels were used to categorize levels of anxiety and depression [43]. The cutoff (> 8) utilized to screen for symptoms for anxiety and depression for each subscale was the same as used previously in the Bangladesh population [44, 46]. In the present study, Cronbach’s alpha of the anxiety and depression subscales were 0.80 and 0.70, respectively, and overall Cronbach’s alpha of the HADS scale was 0.83, indicating good reliability.

### Statistical analysis

Data were analyzed using four statistical software packages including Microsoft Excel 2019, SPSS version 25, SPSS Amos version 23, and STATA version 13. First, data were cleaned, sorted, and coded using Microsoft Excel, and after the completion of data coding, the file was imported to SPSS. Descriptive statistics (i.e., frequencies, percentages, means, standard deviations) were performed using SPSS. Regression analyses with the HADS score as a dependent variable, and all examined variables modeled simultaneously (i.e., adjusted for each other) were executed in STATA. The normally distributed HADS total score was used as the outcome in a multiple linear regression model. The reason for this approach was that the two subscales of depression and anxiety were strongly associated with each other (Pearson *r *= 0.64, *p *< 0.001). Associations were considered statistically significant if the two-sided *p*-value was less than or equal to 0.05. A confirmatory factor analysis (CFA) was performed using SPSS Amos version 23 to evaluate the psychometric properties of the Bangla version of the HADS.

### Ethics

All procedures of the present study were performed in accordance with human involving regulations (i.e., Declaration of Helsinki) and with guidelines of institutional research ethics. The formal ethics approval was granted by the ethical review board of the Faculty of Biological Sciences, Jahangirnagar University, Savar, Dhaka-1342, Bangladesh [BBEC, JU/M 2020 COVID-19/9(5)]. All participants provided their consent to participate in the study after being informed about the purpose of the study. Anonymity and confidentiality of participants’ information were strictly maintained.

## Results

### Participant characteristics

Data from 803 healthcare professionals were included in final analysis. Approximately half (50.7%) were male, mean age was 27.3 years (SD = 6.9; age range: 18-58 years), and most were doctors (68%). Likewise, nurses (10%), public health professionals (7%), lab workers (9%), and other caregivers (6%) participated. Most participants were unmarried (69.9%), resided in urban areas (81.7%), reported good health status (62.4%), and denied chronic diseases (90.2%). Moreover, 52.4% participants reported normal sleep durations, most (62.6%) reported not getting physical exercise, and a minority reported cigarette smoking (13.7%) (Table 1).

**Table 1:** General characteristics of participants (health care workers [HCWs] treating COVID-19 patients; *N *= 803 HCWs)

Variables	*n*	(%)
**Gender**		
Male	407	(50.7)
Female	396	(49.3)
**Age (27.33 ± 6.88)**		
Young (18-25 years)	422	(52.6)
Adult (>25 years)	381	(47.4)
**Marital status**		
Unmarried	561	(69.9)
Married	242	(30.1)
**Occupation**		
Doctors	549	(68.4)
Nurses	77	(9.6)
Public health professionals	59	(7.3)
Lab workers	73	(9.1)
Others caregivers^a^	45	(5.6)
**Residence**		
Rural	147	(18.3)
Urban	656	(81.7)
**Self-reported health status**		
Good	501	(62.4)
Moderate	291	(36.2)
Poor	11	(1.4)
**Chronic diseases**		
Yes	79	(9.8)
No	724	(90.2)
**Sleep status**		
Less than normal	356	(44.3)
Normal (7-9 hours)	421	(52.4)
More than normal	26	(3.2)
**Physical exercising**		
Yes	300	(37.4)
No	503	(62.6)
**Tobacco smoking**		
Yes	110	(13.7)
No	693	(86.3)
**Have you been infected with COVID-19 while at work?**		
Yes	47	(5.9)
No	756	(94.1)
**Have any of your colleagues been infected with COVID-19?**		
Yes	362	(45.1)
No	441	(54.9)
**Do you feel adequately trained to conduct COVID-19-related treatment/PCR tests/provision of patient care?**		
Yes	286	(35.6)
No	517	(64.4)
**Have you experienced shortages of PPE /hand sanitizer/masks during the pandemic?**		
Yes	454	(56.5)
No	349	(43.5)
**Have you had regrets about your choice of profession because of the pandemic and related unexpected experiences?**		
Yes	365	(45.5)
No	438	(54.5)
**Are you frequently updating yourself with the latest research papers on COVID-19?**		
Yes	639	(79.6)
No	164	(20.4)
**Do you currently have to work overtime?**		
Yes	415	(51.7)
No	388	(48.3)
**Have you ever been discriminated against in your workplace (lab or hospital)?**		
Yes	312	(38.9)
No	491	(61.1)
**Have you been counseled regarding how to maintain your mental health in the current situation?**		
Yes	150	(18.7)
No	653	(81.3)
**Have you encountered social problems (e.g., neighbor neglect) due to working in a lab or hospital?**		
Yes	195	(24.5)
No	601	(75.5)
**Anxiety**		
Normal	245	(30.5)
Borderline abnormal	227	(28.3)
Abnormal	331	(41.2)
**Depression**		
Normal	486	(60.5)
Borderline abnormal	191	(23.8)
Abnormal	126	(15.7)

Note: ^a^Pharmacologists, microbiologists, virologists, and biotechnologists

### Psychometric properties of the Bangla HADS

Mean, standard deviation, item-total correlation, Cronbach’s alpha of the scale if each item were omitted, skewness, and kurtosis of each item of the Bangla HADS are presented (Table 2). The inter-item correlation matrix contained no negative values, indicating that the items were assessing the same construct. All items yielded skewness, and kurtosis values within the ± 2.2 range, indicating that they were normally distributed.

**Table 2:** Item-level psychometric properties of the Bangla HADS

Variables	Mean	SD	Skewness	Kurtosis	Item-total correlation	Cronbach's α if Item Omitted
A1	1.47	0.92	0.81	-0.73	0.51	0.82
A2	1.66	0.89	-0.19	-0.70	0.59	0.81
A3	1.45	0.87	0.29	-0.62	0.52	0.82
A4	1.14	0.74	0.29	-0.13	0.49	0.82
A5	0.88	0.72	0.31	-0.64	0.44	0.82
A6	1.67	0.87	-0.19	-0.62	0.52	0.82
A7	1.52	0.86	-0.08	-0.64	0.60	0.81
D1	0.89	0.97	0.79	-0.47	0.46	0.82
D2	0.36	0.70	1.80	2.12	0.32	0.83
D3	1.00	0.68	0.51	0.69	0.59	0.82
D4	1.33	0.87	0.20	-0.62	0.49	0.82
D5	1.77	1.09	-0.43	-1.10	0.20	0.84
D6	0.67	0.83	1.02	0.14	0.43	0.82
D7	0.83	0.89	0.83	-0.13	0.46	0.82

Note: A1: I feel tense or 'wound up'; A2: I get a sort of frightened feeling as if something awful is about to happen; A3: Worrying thoughts go through my mind; A4: I can sit at ease and feel relaxed; A5: I get a sort of frightened feeling like 'butterflies' in the stomach; A6: I feel restless as I have to be on the move; A7: I get sudden feelings of panic; D1: I still enjoy the things I used to enjoy; D2: I can laugh and see the funny side of things; D3: I feel cheerful; D4: I feel as if I am slowed down; D5: I have lost interest in my appearance; D6: I look forward with enjoyment to things; D7: I can enjoy a good book or radio or TV program.

Confirmatory factor analysis (CFA) was performed to evaluate the structural validity of the Bangla HADS instrument using a two-factor model (anxiety, and depression). For this model, the Absolute Fit (i.e., χ^2^, Root Mean Square Error of Approximation [RMSEA], Standardized Root Mean Square Residual [SRMR], Goodness of Fit Index [GFI]), and the Incremental Fit (i.e., Adjusted Goodness of Fit Index [AGFI], Comparative Fit Index [CFI], Tucker-Lewis Index [TLI], Normed Fit Index [NFI]) were observed for the model fit estimation (Table 3). Thresholds and conventional fit indices were applied to investigate the goodness of fit of the model under statistical analysis: RMSEA (< 0.08), SRMR (< 0.08), GFI (> 0.9), AGFI (> 0.90), CFI (> 0.90), TLI (> 0.90), and NFI (> 0.90) [47–50]. All fitness indexes were very satisfactory within their conventional thresholds, suggesting models provided excellent fit to the data.

**Table 3:** Scale-level psychometric properties of the Bangla HADS

Name of index	Index abbreviation	Anxiety subscale	Depression subscale	HADS scale	Level of acceptance
Absolute Fit					
Discrepancy chi square	χ^2^ (df)	52.19* (14)	106.69* (14)	397.18* (76)	*p *> 0.05
Root Mean Square Error of Approximation (90% Confidence interval)	RMSEA (90% CI)	0.06 (0.04-0.08)	0.09 (0.08-0.11)	0.07 (0.07-0.08)	< 0.08
Standardized Root Mean Square Residual	SRMR	0.03	0.06	0.06	< 0.08
Goodness of Fit Index	GFI	0.98	0.96	0.93	> 0.9
Incremental Fit					
Adjusted Goodness of Fit	AGFI	0.96	0.92	0.9	> 0.9
Comparative Fit Index	CFI	0.97	0.9	0.9	> 0.9
Tucker-Lewis Index	TLI	0.96	0.83	0.9	> 0.9
Normed Fit Index	NFI	0.96	0.9	0.9	> 0.9

Note: *p < 0.001

**Fig. 1:**
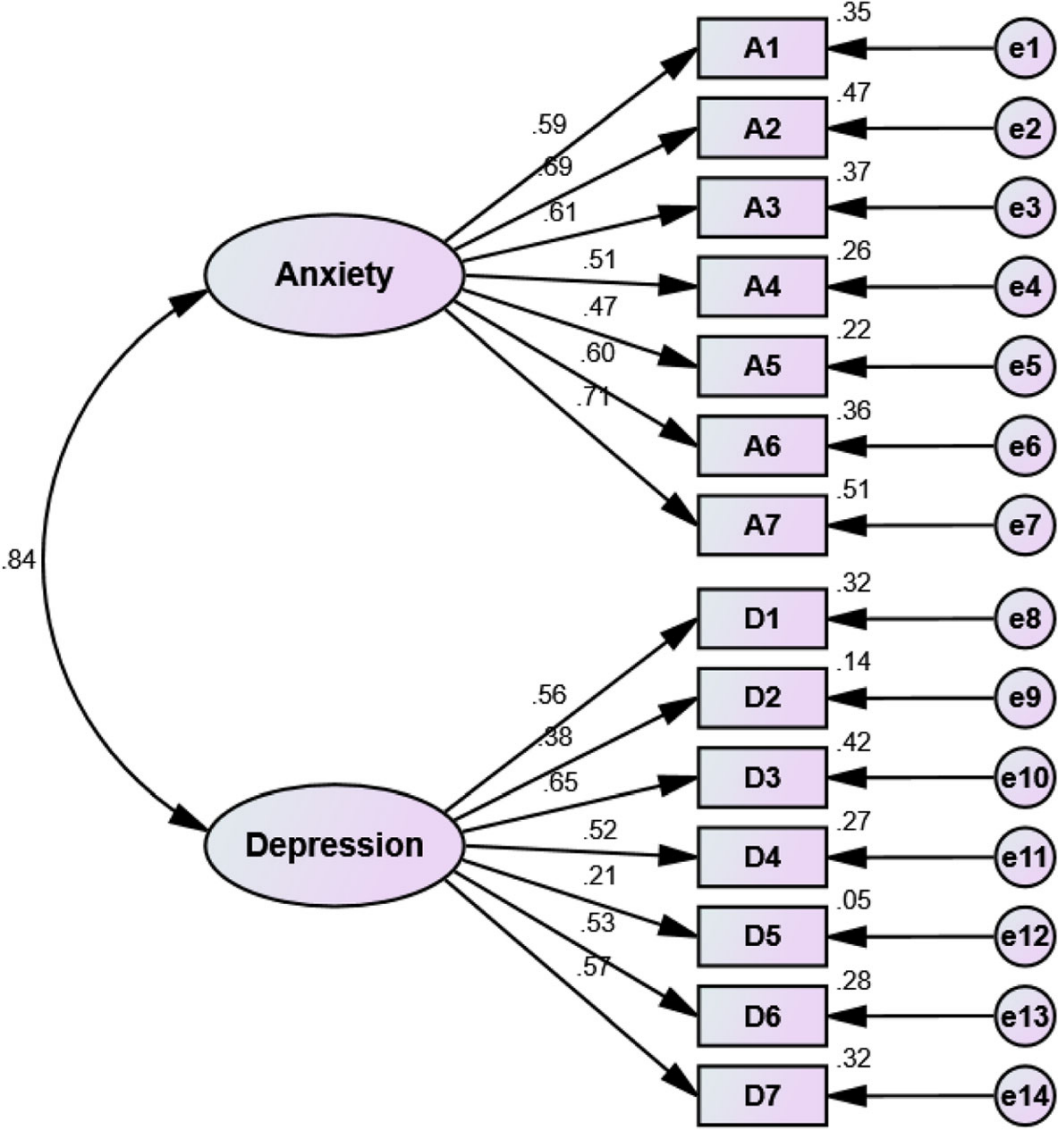
Structural equation modeling (SEM) of the Bangla Hospital Anxiety and Depression Scale (HADS). Note: Items of anxiety subscale (A1, A2, A3, A4, A5, A6, A7); Items of Depression subscale (D1, D2, D3, D4, D5, D6, D7); All e values represent (unobserved variables). The specific questions to which these items refer are as follows. A1: I feel tense or 'wound up'; A2: I get a sort of frightened feeling as if something awful is about to happen; A3: Worrying thoughts go through my mind; A4: I can sit at ease and feel relaxed; A5: I get a sort of frightened feeling like 'butterflies' in the stomach; A6: I feel restless as I have to be on the move; A7: I get sudden feelings of panic; D1: I still enjoy the things I used to enjoy; D2: I can laugh and see the funny side of things; D3: I feel cheerful; D4: I feel as if I am slowed down; D5: I have lost interest in my appearance; D6: I look forward with enjoyment to things; D7: I can enjoy a good book or radio or TV program

Figure 1 presents structural equation modeling (SEM) of the Bangla HADS, and demonstrates that the two subscales (anxiety, and depression) were positively and significantly correlated with each other. The factor loadings for each item of the Bangla HADS ranged between 0.38 and 0.71 (except item D5), and were acceptable. The acceptability factor is greater than the load value of 0.32 [51].

### Symptoms at different severity levels

The HADS’ anxiety subscale revealed that anxiety symptoms were experienced by HCWs at different predetermined thresholds including normal (30.5%), borderline abnormal (28.3%), and abnormal (41.2%) levels. Regarding the depression subscale, symptoms were experienced by HCWs at normal (60.5%), borderline abnormal (23.8%), and abnormal (15.7%) levels. The anxiety and depression scores were strongly associated with each other (Pearson *r *= 0.64).

### Regression analyses

The HADS-score was significantly (*p *< 0.05) higher among participants who reported being female, having moderate and poor health status, not engaging in physical exercising, smoking tobacco, having had regrets about their profession because of the pandemic and related unexpected experiences, not updating on the latest COVID-19-related research, experiencing discrimination in the workplace, and facing social problems due to working in a lab or hospital (Table 4).

**Table 4:** Regression analyses by total HADS score with all examined variables

Variables	Total HADS score			
	***Mean***	***(SD)***	***β***	***SE***
**Gender**				
Male	16.1	(6.8)	^a^	
Female	17.2	(6.6)	0.13***	0.43
**Age**				
Young (18-25 years)	17.0	(6.6)	^a^	
Adult (>25 years)	16.2	(6.8)	-0.02	0.52
**Marital status**				
Unmarried	16.7	(6.5)	^a^	
Married	16.4	(7.2)	-0.06	0.56
**Residence**				
Rural	16.0	(7.2)	^a^	
Urban	16.8	(6.6)	0.03	0.53
**Self-reported health status**				
Good	14.7	(6.0)	^a^	
Moderate	19.4	(6.4)	0.23***	0.46
Poor	29.0	(6.7)	0.18***	1.79
**Chronic diseases**				
No	16.3	(6.6)	^a^	
Yes	19.3	(7.1)	0.04	0.74
**Sleep status**				
Less than normal	16.6	(6.4)	^a^	
Normal (7-9 hours)	16.5	(6.8)	-0.05	0.45
More than normal	18.2	(9.8)	-0.02	1.18
**Physical exercising**				
Yes	14.6	(6.3)	^a^	
No	17.8	(6.7)	0.08**	0.44
**Tobacco smoking**				
No	16.4	(6.6)	^a^	
Yes	18.0	(7.4)	0.07*	0.64
**Have you been infected with COVID-19 while at work?**				
No	16.5	(6.7)	^a^	
Yes	18.0	(7.1)	-0.02	0.88
**Have any of your colleagues been infected with COVID-19?**				
No	15.8	(6.4)	^a^	
Yes	17.7	(7.0)	0.03	0.45
**Do you feel adequately trained to conduct COVID-19-related treatment/PCR tests/provision of patient care?**				
Yes	16.0	(6.2)	^a^	
No	17.0	(7.0)	-0.01	0.48
**Have you experienced shortages of PPE /hand sanitizer/masks during the pandemic?**				
No	15.8	(6.2)	^a^	
Yes	17.2	(7.0)	-0.04	0.45
**Have you had regrets about your choice of profession because of the pandemic and related unexpected experiences?**				
No	14.6	(6.0)	^a^	
Yes	19.0	(6.7)	0.16***	0.46
**Are you frequently updating yourself with the latest research papers on COVID-19?**				
Yes	16.0	(6.4)	^a^	
No	19.2	(7.3)	0.08**	0.52
**Do you currently have to work overtime?**				
No	16.5	(6.9)	^a^	
Yes	16.7	(6.6)	-0.03	0.46
**Have you ever been discriminated against in your workplace (lab or hospital)?**				
No	14.8	(6.1)	^a^	
Yes	19.5	(6.6)	0.21***	0.47
**Have you been counseled regarding how to maintain your mental health in the current situation?**				
Yes	14.7	(5.1)	^a^	
No	17.1	(7.0)	-0.01	0.62
**Have you encountered social problems (e.g., neighbor neglect) due to working in a lab or hospital?**				
No	15.6	(6.4)	^a^	
Yes	19.7	(6.8)	0.13***	0.51

Note:

*SE* Standard error

*β*– Standardized regression coefficient

*p < 0.05, **p < 0.01, ***p < 0.001

^a^ Reference category

## Discussion

The COVID-19 pandemic appears to have impacted the lives of many individuals, and may influence mental wellbeing [37], especially among HCWs who are on the frontline caring for people with COVID-19. To our best knowledge, the present study is the first to assess anxiety and depression among HCWs in Bangladesh during the COVID-19 pandemic. Our hypotheses were partially supported in that anxiety and depression were prevalent among HCWs, and some but not all COVID-19-related experiences were linked to anxiety and depression. The study also investigated the psychometric properties of the Bangla HADS. The Bangla HADS was psychometrically sound, and demonstrated good internal consistency and reliability (α = 0.83), and excellent construct validity. The study included doctors, nurses, public health professionals, lab workers, and other caregivers (e.g., pharmacologists, microbiologists, virologists, biotechnologists) who were directly or indirectly involved in the care of patients with COVID-19.

### Comparisons with other studies

The current findings indicate that anxiety and depression are common among HCWs in Bangladesh during the COVID-19 pandemic, with 69.5% screening positive for anxiety, and 39.5% for depression. A cross-sectional study across 34 hospitals in China similarly found that frontline HCWs experienced mental health concerns including depression and anxiety when directly engaged with treating and managing patients with COVID-19 [10]. A prior study in China documented prevalence estimates of anxiety (46.0% versus 39.5% in the present study) with the Generalized Anxiety Disorder Scale (GAD-7) and depression (44.4% versus 69.5% in the present study) with the Patient Health Questionnaire (PHQ-9) among HCWs during the COVID-19 pandemic [7], which suggests that the prevalence estimate of anxiety was somewhat higher and that of depression lower compared to the present study. Another study of anxiety and depression among first-line medical staff in China showed prevalence estimates of anxiety (using Self-Rating Anxiety Scale) and depression (using Self-Rating Depression Scale) of 45.6%, and 11.4%, respectively [52]. A study in India of HCWs suggested the prevalence estimates of anxiety symptoms (17.7%) requiring additional evaluation and depressive symptoms (11.4%) needing treatment [53] are lower than those in the present study. Moreover, an examination of anxiety that included 12 studies (1 from Fuyang, 2 Wuhan, 1 Fujian, 1 Singapore, 7 from China), and for depression that included 10 studies (2 Wuhan, 1 Fujian, 1 Singapore, 6 from China) reported that the pooled prevalence estimates of anxiety and depression among HCWs during COVID-19 were 23.2% and 22.8%, respectively, and these values are numerically lower than those in the present study [54]. However, one should consider comparisons across studies cautiously given other factors that may influence estimates, and studies that directly compare estimates concurrently across jurisdictions, and using similar methodologies are warranted. The extent to which different findings may relate to cultural differences, differences in healthcare systems, and their responses during the COVID-19 pandemic, differences in instruments used to assess anxiety and depression or other factors warrants additional investigation.

Females were more likely to experience anxiety and depression, similar to prior studies conducted in Bangladesh during the COVID-19 pandemic [39, 55]. However, the findings contrast with those from a study among university students that showed no gender-related differences [56]. A prior study conducted among Chinese medical staff during the COVID-19 pandemic reported that anxiety was more frequently observed in females relative to males [57].

HCWs with moderate and poor health status were more depressed and anxious than those who self-reported good health. These findings resonate with those among Bangladeshi university students in which individuals with moderate and poorer self-related health were more depressed and anxious [58]. Another study conducted in Bangladesh among individuals who had recovered from COVID-19 found a positive association between poor health status and depression [59]. The findings are also similar to those from a study in Syria among community adults that observed an association between depression and chronic diseases including heart disease, hypertension, and kidney disease [60], and findings from a literature review [61].

In the present study, participants who did not engage in physical exercising compared to those who did were more likely to endorse anxiety and depression, similar to prior studies conducted in Bangladesh [39, 56, 59, 62]. HCWs who smoked cigarettes were more likely to be anxious and depressed than non-smokers, also consistent with previous studies [63–66]. A pre-COVID-19 study among medical staff in Greece similarly showed significant associations with anxiety and depression [67]. These findings may have clinical relevance as exercise and smoking represent modifiable risk factors. Therefore, if the associations between low exercise and/or smoking, and poor mental health are causal [68–70], risk reduction strategies focusing on regular exercise and smoking discontinuation may be helpful in reducing potentially deleterious impacts of COVID-19 on the mental health of HCWs. In such efforts, combination approaches may be particularly relevant [71]; however, other stress-reduction approaches (e.g., meditation, massage) may be helpful, although more research is needed in this area [72]. Further, some benefits of engaging in physical activity may be transmitted to patients, although this too warrants further study [73].

Anxiety and depression were associated with negative feelings about choice of profession due to the ongoing crisis of the pandemic and unexpected pandemic-related experiences. Considering the limited availability of PPE, disinfection materials, and medical (N95) masks, and increasing numbers of positive COVID-19 cases [74], HCWs may question their choice of profession. Other factors (e.g., possible trauma-related to patient care during the pandemic) may also impact HCWs, and lead to mental health concerns.

Anxiety and depression were significantly higher among respondents who did not feel up to date on the latest COVID-19-related research/information. Lack of information may precipitate mental health concerns, and prior studies have suggested updates and knowledge about COVID-19 may have psychosocial impacts, possibly as they represent an active way of coping and dealing with pandemic-related issues [75].

HCWs who encountered discrimination in their workplace were more likely to be anxious, similar to findings among Chinese physicians in which workplace violence was associated with poor mental health [76]. Other studies have found that discrimination in the workplace was related negatively to mental wellbeing [77–79]. Given the current scarcity of HCWs in Bangladesh, shortages of PPE and fear of being infected by the virus [74], and transmitting it to family members could promote anxiety and depression. As a lower-middle income country, Bangladesh is particularly vulnerable to the transmission of a communicable disease due to overcrowded environments, and having poor infection prevention and control mechanisms [74]. As such, HCWs may fear that they may infect their families, particularly in Bangladesh because of multifamily households, which often do not permit separate personal spaces. These issues may pose particular challenges for Bangladeshi HCWs during the pandemic. A previous study reported frequent reports of lack of water (50%), and handwashing soaps used to clean hands and kill germs/coronavirus (39%) in health care facilities in low-middle income countries [80]. HCWs should be provided with adequate PPE to protect themselves and their patients within a health-promoting atmosphere.

Respondents facing social problems due to their profession were more likely to experience anxiety and depression. Similarly, social difficulties and stressful incidents have been linked to anxiety and depression [81].

The present study revealed that anxiety and depression were strongly associated with each other (*r *= 0.64). Anxiety and depression frequently co-occur [82], including in Bangladeshi groups [39, 56, 66, 83].

### Implications

The present findings indicate that many Bangladeshi HCWs have been experiencing anxiety and/or depression during the COVID-19 pandemic. Multiple factors may contribute to anxiety and depression, including those inside and outside of workplace settings. After severe crises have ended, anxiety and depression may persist, and PTSD may emerge [12, 18]. The present findings suggest that understanding factors influencing the mental health of HCWs, and those that may generate vulnerability or promote resilience are important to understand currently and moving forward. It seems important to implement monitoring initiatives, and possible interventions to help potentially vulnerable groups like HCWs.

### Limitations

This study has several limitations. First, it was a cross-sectional online survey involving a convenience sample. Hence, causal inferences cannot be defined. Further studies are required to examine longitudinal trajectories of anxiety and depressive symptoms in HCWs during and following the COVID-19 pandemic. Second, there was a small number of doctors, nurses, lab workers, and public health professionals who participated, which may not be representative of the general population. Finally, findings of self-reported mental health symptomatology may differ from those obtained from clinical interviews.

## Conclusions

The present study describes prevalence estimates and correlates of anxiety and depression in HCWs during the COVID-19 pandemic in Bangladesh. HCWs’ mental wellbeing is an important consideration during the COVID-19 outbreak. Interventions to promote the mental wellbeing of HCWs during and following the COVID-19 pandemic should be introduced, implemented, and tested, with a particular focus on vulnerable groups.

## Data Availability

The datasets used and/or analyzed during the current study are available from the corresponding author on reasonable request.

## Abbreviations

HCWs: Healthcare workers
HADS: Hospital Anxiety and Depression Scale
SARS CoV-2: Severe acute respiratory syndrome coronavirus-2
COVID-19: Coronavirus disease-2019
WHO: World Health Organization
PPE: Personal protective equipment
PTSD: Post-traumatic stress disorder
CFA: Confirmatory factor analysis
RMSEA: Root Mean Square Error of Approximation
SRMR: Standardized Root Mean Square Residual
GFI: Goodness of Fit Index
AGFI: Adjusted Goodness of Fit Index
CFI: Comparative Fit Index
TLI: Tucker-Lewis Index
NFI: Normed Fit Index
